# Structural changes in the collagen network of joint tissues in late stages of murine OA

**DOI:** 10.1038/s41598-022-13062-y

**Published:** 2022-06-01

**Authors:** Natalie K. Yoshioka, Gregory M. Young, Deepak Kumar Khajuria, Vengadeshprabhu Karuppagounder, William J. Pinamont, Julie C. Fanburg-Smith, Thomas Abraham, Reyad A. Elbarbary, Fadia Kamal

**Affiliations:** 1 Center for Orthopedic Research and Translational Sciences (CORTS), Penn State College of Medicine, Hershey, PA USA; 2 Department of Orthopedics and Rehabilitation, Penn State College of Medicine, Hershey, PA USA; 3 Department of Pathology, Penn State Health/Milton S. Hershey Medical Center, Hershey, PA USA; 4 Department of Neural and Behavioral Science, Penn State University College of Medicine, Hershey, PA USA; 5 Microscopy Imaging Facility, Penn State University College of Medicine, Hershey, PA USA; 6 Department of Biochemistry and Molecular Biology, Penn State College of Medicine, Hershey, PA USA; 7 Department of Pharmacology, Penn State College of Medicine, Hershey, PA USA

**Keywords:** Structural biology, Diseases

## Abstract

Osteoarthritis (OA) is the most prevalent degenerative joint disease, resulting in joint pain, impaired movement, and structural changes. As the ability of joint tissue to resist stress is mainly imparted by fibrillar collagens in the extracellular matrix, changes in the composition and structure of collagen fibers contribute to the pathological remodeling observed in OA joints that includes cartilage degeneration, subchondral bone (SCB) sclerosis, and meniscal damage. Using the established OA model of destabilization of the medial meniscus (DMM) in C57BL/6J mice, we performed a comprehensive analysis of the content and structure of collagen fibers in the articular cartilage, subchondral bone, and menisci using complementary techniques, which included second harmonic generation microscopy and immunofluorescence staining. We found that regions exposed to increased mechanical stress in OA mice, typically closest to the site of injury, had increased collagen fiber thickness, dysregulated fiber formation, and tissue specific changes in collagen I and II (Col I and Col II) expression. In cartilage, OA was associated with decreased Col II expression in all regions, and increased Col I expression in the anterior and posterior regions. Col I fiber thickness was increased in all regions with disorganization in the center region. In the superficial SCB, all regions exhibited increased Col I expression and fiber thickness in OA mice; no changes were detected in the deeper regions of the subchondral bone except for increased Col I fiber thickness. In the menisci, OA led to increased Col I and Col II expression in the vascular and avascular regions of the anterior meniscus with increased Col I fiber thickness in these regions. Similar changes were observed only in the vascular region of the posterior meniscus. Our findings provide, for the first time, comprehensive insights into the microarchitectural changes of extracellular matrix in OA and serve as guidelines for studies investigating therapies that target collagenous changes as means to impede the progression of osteoarthritis.

## Introduction

Osteoarthritis (OA) is the most prevalent degenerative joint disease and is characterized by limited mobility and impaired joint function. The ability of joints to withstand stress is affected by factors such as age, injury, obesity, and genetics, which can predispose individuals to develop OA^1–4^. Although OA has been initially classified as a disease of articular cartilage degeneration, recent studies have demonstrated the impact of OA on other joint tissues. Accordingly, subchondral bone sclerosis, osteophyte formation, synovitis, and meniscal tears have emerged as hallmarks for OA development and progression^3–6^. Crucially, altered composition of the extracellular matrix (ECM) in any of the joint tissues is expected to impact the functionality and structural integrity of the whole joint. Type I and Type II collagen are the most abundant proteins in the ECM of bone and cartilage, respectively^7^, while menisci are fibrocartilaginous, with an ECM that is also composed of both type I and II collagen^8,9^. Type I and II collagens are both fibrillar collagens, meaning their collagen molecules are cross-linked into fibrils, which self-assemble into fibers^10^. Collagen fibers preserve the structure of the tissues by resisting deformation^11^, allowing the tissue to withstand stress. Examination of the collagen network in the ECM during OA progression is therefore crucial, as it provides valuable insights into the causes that underpin OA-associated loss of joint structural integrity. To characterize the collagenous changes of the ECM in OA joint tissues, we used second harmonic generation (SHG) microscopy to image anterior, center, and posterior regions of the medial knee compartment, defining alterations in not only articular cartilage, but subchondral bone and menisci as well.

SHG imaging is a powerful, label-free method for visualizing fibrillar collagens^12,13^. During the SHG process, samples are exposed to two photons of equal wavelength, which combine and are emitted as either forward (FSHG) or backward (BSHG) signal if the wavelength passes through the structure or is reflected off the structure. Type I collagen typically produces a strong FSHG signal, while Type II collagen typically produces a strong BSHG signal. While other fibrillar collagens (Col III, V, XI, XXIV, XXVII) are present in the joint tissues, they generate weak SHG signals^14,15^. FSHG and BSHG signals can then be analyzed to determine fiber content, width, and orientation. SHG has been utilized to examine collagenous changes in articular cartilage of early stage OA^16^. SHG is also emerging as a promising in-vivo-imaging application for diagnostic evaluation of collagenous changes in articular cartilage and menisci^17,18^.

Previous studies have examined the relationship of joint tissue degeneration between cartilage, subchondral bone, and menisci^19^, but they either explored structural changes to the collagenous network in one tissue type^20–22^, or described singular parameters of the collagen network^20,22^. Kinematic studies of OA knees have shown that changes in distribution of joint loading result in regional changes in the tibial plateau, leading to degenerative changes in the medial compartment and varus deformities^23,24^. To our knowledge, no previous studies have provided a detailed account of the regional changes in collagenous structures in different tissues of late-stage OA knee joints. Here, we provide a comprehensive analysis of the pathological changes in the structural organization and composition of collagen fibers in the different tissues of knee joints in late-stage OA.

## Results

### Microarchitectural changes are evident in tissues of the medial compartment of late-stage OA knee joints

**Figure 1:**
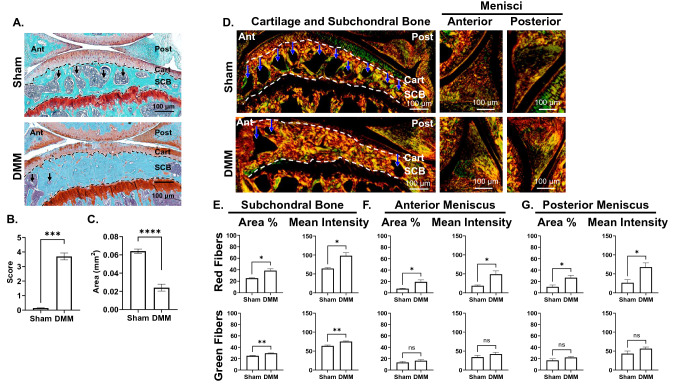
Picrosirius red staining of the knee joint shows changes in the microarchitecture of knee joint tissues in OA mice. (**A**) Representative Safranin-O/Fast Green staining for comparison of Sham and DMM knee joints (× 10 magnification, scale bar = 100 μm, images acquired with OsteoMeasure software) Ant = anterior, Post = posterior, Cart = cartilage, SCB = subchondral bone. Black arrows indicate marrow space in the SCB. (**B**) Osteoarthritis cartilage histopathology assessment system (OARSI) scoring for samples analyzed (0 = no OA activity seen, stage 0, 5 = > 50% involvement OA, stage 4). (**C**) Uncalcified tibial cartilage area (UTC) in mm^2^ calculated by subtracting calcified cartilage area (cartilage between chondro-osseous junction and tideline) from total cartilage area (total articular cartilage above the chondro-osseous junction). (**D**) Representative images of PSR for a comparison of the collagenous structure in cartilage, subchondral bone, and anterior/posterior menisci. Thicker fibers typically stain red while thinner fibers stain green. Blue arrows indicate marrow space in the SCB (× 10 magnification, scale bar = 100 μm). PSR staining quantification of the area percentage and mean intensity of red and green fibers in the (**E**) subchondral bone, (**F**) anterior meniscus, and (**G**) posterior meniscus. *P < 0.05, **P < 0.01, ***P < 0.001, ****P < 0.0001 using unpaired t-test with Welch’s correction, values are expressed as mean ± SEM; N = 5/group.

12 weeks following DMM, mice knees showed loss of articular cartilage, decreased bone marrow space (arrows, Fig. 1), and increased sclerosis in the subchondral bone (Fig. 1A–C), as we have previously showed^1^. For a preliminary evaluation of the collagen microarchitecture, we stained knee joints of both sham and DMM-operated mice with Picrosirius red (PSR)^2,3^, in which samples were imaged with a polarized light microscope to selectively image collagen fibers (Fig. 1D). Red birefringence indicates thicker fibers, while green birefringence indicates thinner fibers^3,25^. In the cartilage, DMM mice had reduced uncalcified cartilage area (Fig. 1C) with a trend towards reduced area and intensity of the green birefringence (Fig. S1A). This green birefringence originates from the radial collagen network, which is composed of thin fibers mainly formed by Col II, cross-linked with minor fibrillar collagens like Col III, V, and XI^26,27^. In the subchondral bone, DMM mice showed decreased marrow space (blue arrows, Fig. 1D), with increase in both red and green birefringence area and intensity (Fig. 1E). In both anterior and posterior menisci, DMM mice had a significant increase in red birefringence area and intensity with only a trend towards increase in green birefringence area and intensity (Fig. 1F,G). These results indicated reduction in uncalcified tibial cartilage and increased collagen fiber deposition in the subchondral bone and both the anterior and posterior menisci (Fig. S1A, Fig. 1E–G). We used these results as a preliminary guide to confirm that OA progression affects the collagenous structure of not only cartilage, but also SCB and menisci. Although PSR is useful for quick analysis of tissue remodeling, PSR cannot be used to distinguish different collagen types^25^. Thus, we performed the regional analysis of the medial knee-joint compartment with IF staining and SHG imaging for more comprehensive specific analysis of fibrillar collagen changes.

### OA results in substantial changes in the microstructure of collagen fibers in the articular cartilage

**Figure 2:**
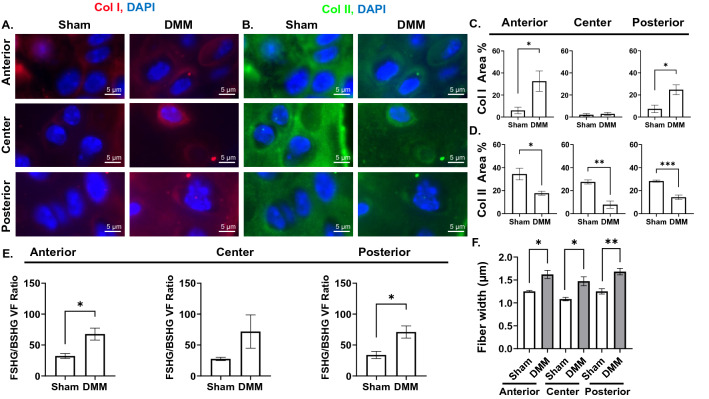
OA results in changes to collagen content, fiber width, and organization in the articular cartilage. (**A**) Immunofluorescence (IF) staining for Col I and (**B**) Col II expression in anterior, center, and posterior regions of articular cartilage, (× 40 magnification, scale bar = 5 μm, images acquired with Zeiss Zen Blue 2.6 edition software). Quantification of area percentage (area of positive signal normalized to total selected area) of (**C**) Col I and (**D**) Col II IF stains. (**E**) Volume fraction (volume producing SHG signal in the selected ROI normalized to the total volume) ratio between FSHG/BSHG VF in ROIs of articular cartilage. (**F**) Comparison of FSHG fiber width (μm) between sham and DMM samples in each ROI. *P < 0.05, **P < 0.01, ***P < 0.001 using unpaired t-test with Welch’s correction, values are expressed as mean ± SEM; 3 images were analyzed per ROI in each sample, N = 5/group.

First, to analyze how the expression of Col I and Col II changes in OA tibial cartilage, we performed immunofluorescence (IF) staining of Col I and Col II in sham and DMM mice and analyzed the area % and average intensity of the expression of both proteins in the anterior, center, and posterior regions of the cartilage (Fig. 2A–D). Because the superficial and transitional zones are degraded in late-stage OA, we imaged the remaining radial zone in the uncalcified cartilage. Results indicated that Col I expression area was very low in the sham cartilage, but significantly increased in the anterior and posterior regions of the cartilage of DMM mice (Fig. 2C), with increased average intensity in the anterior region (Fig. S1B). This increase in Col I expression in DMM mice was accompanied by a significant reduction in Col II expression area in all analyzed regions (Fig. 2D) and unchanged average intensity (Fig. S1B). This reduction in Col II expression is consistent with cartilage degeneration (Fig. 1A–C). Supporting these results, SHG analysis of FSHG signal, which is strongly produced by Col I fibers, and BSHG signal, which is produced by both Col I and Col II fibers, demonstrated a significant increase in FSHG/BSHG volume fraction ratio in the anterior and posterior regions of the tibial cartilage (Fig. 2E). We then analyzed the microstructure of collagen fibers. To avoid confounding the data by analyzing the BSHG signal that is generated by both Col I and Col II fibers which generally colocalize in OA cartilage, we focused our analysis on the FSHG signal (i.e. Col I fibers). Results indicated significant thickening of FSHG-producing fibers in all regions of DMM cartilage (Fig. 2F). Further, to characterize how OA pathology affects the arrangement Col I fibers, we computed the orientation index in all regions of the articular cartilage. The central region exhibited less organized FSHG-producing fibers in DMM samples as compared to sham samples (Fig. S1C). Interestingly, in sham mice, collagen fiber arrangement was significantly more oriented in the central region compared to the anterior region (Fig. S1D), whereas no significant difference in fiber orientation was noted among different regions of DMM cartilage (Fig. S1E).

### OA results in increased expression of Col I and thickness of Col I fibers in the SCB

**Figure 3:**
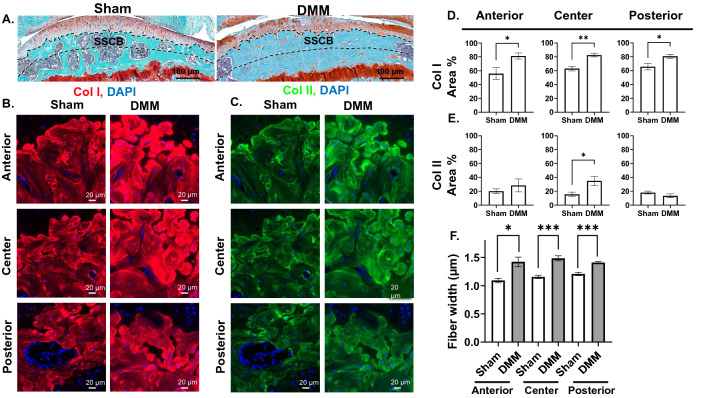
OA results in increased collagen composition and increased fiber thickness in the superficial regions of subchondral bone. (**A**) Safranin-O/Fast Green staining depicting the superficial SCB (SSCB) (10 × magnification, scale bar = 100 μm, images acquired with OsteoMeasure software). IF staining for (**B**) Col I and (**C**) Col II expression in anterior, center, and posterior regions of the superficial SCB (× 40 magnification, scale bar = 20 μm, images acquired with Zeiss Zen Blue 2.6 edition software). Quantification of area percentage (area of positive signal normalized to total selected area) of (**D**) Col I and (**E**) Col II IF stains. (**F**) Comparison of FSHG fiber width (μm) between sham and DMM samples in each ROI. *P < 0.05, **P < 0.01, ***P < 0.001 using unpaired t-test with Welch’s correction, values are expressed as mean ± SEM; 3 images were analyzed per ROI in each sample, N = 5/group.

**Figure 4:**
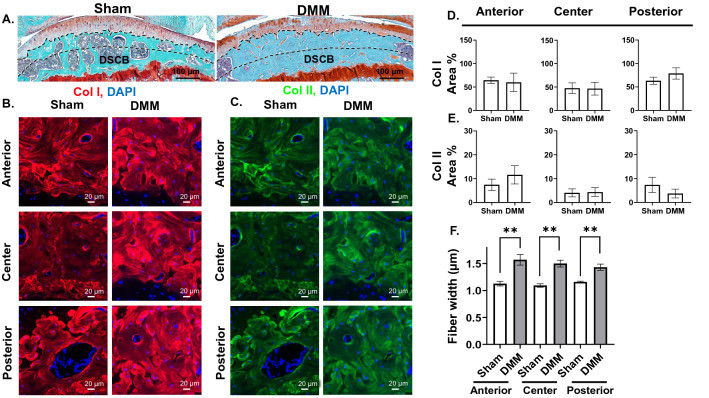
OA results in increased fiber thickness in the deep regions of subchondral bone. (**A**) Safranin-O/Fast Green staining depicting the deep SCB (DSCB) (× 10 magnification, scale bar = 100 μm, images acquired with OsteoMeasure software). IF staining for (**B**) Col I and (**C**) Col II expression in anterior, center, and posterior regions of deep SCB (× 40 magnification, scale bar = 20 μm, images acquired with Zeiss Zen Blue 2.6 edition software). Quantification of area percentage (area of positive signal normalized to total selected area) of (**D**) Col I and (**E**) Col II IF stains. (**F**) Comparison of FSHG fiber width (μm) between sham and DMM samples in each ROI. **P < 0.01 using unpaired t-test with Welch’s correction, values are expressed as mean ± SEM; 3 images were analyzed per ROI in each sample, N = 5/group.

PSR indicated increased collagen fiber thickness illustrated by an increase in red-staining fibers in sclerotic SCB (Fig. 1E) and reduced marrow space in DMM samples (blue arrows, Fig. 1D). To further investigate this, we analyzed collagen fibers in both the superficial (close to the articular surface) and deep regions (close to the growth plate) of SCB in sham and DMM mice (labeled SSCB and DSCB respectively in Figs. 3A, 4A). IF staining indicated significant and consistent elevation in Col I expression area in all regions of the superficial, but not the deep, SCB of DMM mice (Figs. 3B,D, 4B,D), with unchanged average intensity (Fig. S2A,B). FSHG-producing fibers (Col I) were also consistently thicker than normal in all regions of both the superficial and deep zones (Figs. 3F, 4F). No change in the orientation of FSHG-producing fibers was observed in any of the analyzed regions of the superficial or deep SCB (Fig. S2C,D). Interestingly, Col II expression area increased in the central region of the superficial SCB (Fig. 3C,E), with no change in any of the deep SCB regions (Fig. 4C,E). Col II average intensity remained unchanged throughout all regions in both superficial and deep SCB (Fig. S2A,B). FSHG/BSHG VF ratio was not calculated for a measure of expression of Col I relative to Col II in the SCB, as IF staining showed that their expression increased simultaneously.

### OA is accompanied by changes in collagen composition and fiber thickness in the menisci

**Figure 5:**
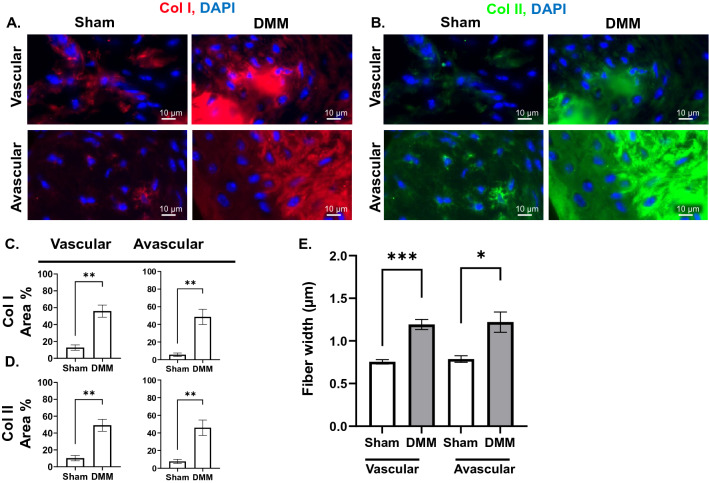
OA results in changes to collagen composition and fiber thickness in the anterior meniscus. IF staining for (**A**) Col I and (**B**) Col II expression in vascular (peripheral) and avascular (internal) regions of the anterior meniscus (× 40 magnification, scale bar = 10 μm, images acquired with Zeiss Zen Blue 2.6 edition software). Quantification of area percentage (area of positive signal normalized to total selected area) of (**C**) Col I and (**D**) Col II IF stains. (**E**) Comparison of FSHG fiber width (μm) between sham and DMM samples in each ROI. *P < 0.05, **P < 0.01, ***P < 0.001 using unpaired t-test with Welch’s correction, values are expressed as mean ± SEM; 3 images were analyzed per ROI in each sample, N = 5/group.

**Figure 6:**
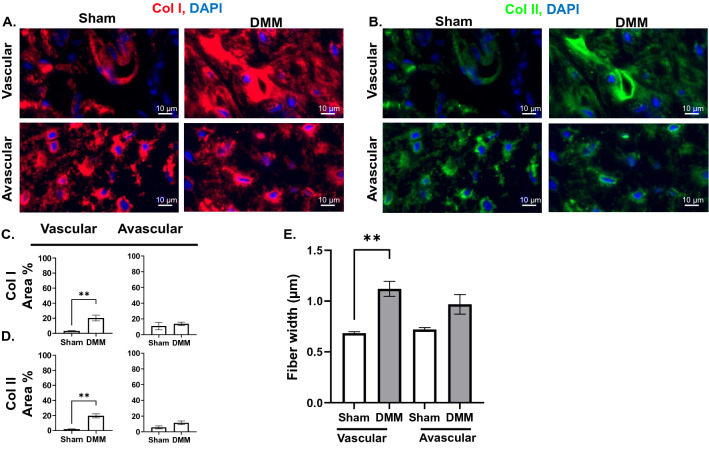
Collagen composition and fiber thickness in the posterior meniscus are affected by OA. IF staining for (**A**) Col I and (**B**) Col II expression in vascular (peripheral) and avascular (internal) regions of the posterior meniscus (× 40 magnification, scale bar = 20 μm, images acquired with Zeiss Zen Blue 2.6 edition software). Quantification of area percentage (area of positive signal normalized to total selected area) of (**C**) Col I and (**D**) Col II IF stains. (**E**) Comparison of FSHG fiber width (μm) between sham and DMM samples in each ROI. **P < 0.01 using unpaired t-test with Welch’s correction, values are expressed as mean ± SEM; 3 images were analyzed per ROI in each sample, N = 5/group.

Increased vascularization of the menisci is known to occur after knee injury, resulting in calcification and chondrocyte hypertrophy^28,29^. Therefore, we assessed changes in collagen composition in the vascular (outer 10–30% of the meniscus) and avascular (inner 70–90% of the meniscus) regions^30^ of both the anterior and posterior menisci. In the anterior meniscus of DMM mice, the expression area of both Col I and Col II was significantly increased in both the vascular and avascular compartments (Fig. 5A–D), with unchanged average intensity (Fig. S3A). The width of the FSHG-producing fibers was increased in both regions (Fig. 5E). In the posterior meniscus of DMM mice, similar elevations in the expression area of Col I and Col II (Fig. 6A–D), and width of FSHG-producing fibers (Fig. 6E) were observed in the vascular, but not the avascular; with unchanged average intensity (Fig. S3B). No change in the FSHG or BSHG OI was observed in any region of either the anterior or posterior menisci (Figs. S3C–D). As both Col I and II expression increased in the avascular and vascular regions of meniscal horns, FSGH/BSGH VF ratio was not calculated.

## Discussion

We present a comprehensive analysis of the microstructural changes that occur in collagen fibers in late OA stages, which leads to the structural remodeling of the knee joint. Previous studies of the collagenous network in human knee joints have found that OA progression results in collagenous disorganization of the posterior meniscal horn^21^ and misaligned, thickened fibers in the superficial and deep zones of the articular cartilage^31^, which contribute to structural changes leading to tissue degradation. Our study extends the scope of previous studies that focused on examining the initial changes that occur in the cartilage during early OA^4,16,32^, or singular joint tissues^21^. Here, we define regional differences in the medial compartment of the knee in late OA, which provides indication of how load is being re-distributed in later stages of the disease. Furthermore, as OA is known to impact not only cartilage, but also multiple joint tissues including subchondral bone and menisci, we provide a comprehensive characterization of OA-associated collagenous changes in different regions and tissues composing the knee joint.

Late-stage OA is characterized by extensive cartilage degeneration and increased Col I expression that mediates mineralization of the articular cartilage^16,33^. Our comprehensive analyses of fibrillar collagen using complementary PSR staining, IF staining, and SHG techniques confirm that the uncalcified region of DMM mice exhibits reduced expression of Col II protein, loss of Col II perpendicular collagen fibers in the radial zone, as well as pathologically elevated expression of Col I (Fig. 2A–E). Regionally, Col I expression increases in the anterior and posterior, but not the central, regions of the DMM articular cartilage (Fig. 2A,C,E). This non-uniform pattern of increased expression can be attributed to changes in the joint structure that accompany OA and altered force distribution to create more loading toward the periphery, as opposed to even force distribution across the tibial plateau in normal knees.

Previous SHG analysis of early stage OA showed that collagen I and II fibers in the radial zone of the cartilage become thinner and more organized^16^. Here, we show that as OA proceeds to late stage, Col I fibers undergo thickening in all regions of the cartilage (Fig. 2F) and become markedly disorganized in the central region (Fig. S1C). In general, the central region of intact cartilage experiences more direct contact with the femur in comparison to other regions of the tibial plateau due to the absence of meniscal fibrocartilage protection^34^, resulting in an increasingly displaced collagenous network^34^ that undergoes re-orientation when exposed to stress^35,36^. Therefore, the observed fiber re-orientation in this region of OA cartilage (Fig. S1C) might act, together with fiber thickening, as a compensatory mechanism for the lost support and the resultant increased mechanical loads^35,36^.

As crosstalk between the SCB and the cartilage impacts chondrocytes homeostasis and OA progression^5,37^, understanding the SCB structural changes in late-stage OA is crucial for uncovering the underlying mechanisms contributing to cartilage loss with OA progression. The SCB is separated from the calcified cartilage by the chondro-osseous junction and is composed of the SCB plate and trabecular portion^3,5,38,39^. The SCB plate resists stress and undergoes dynamic re-orientation in response to environmental stimuli and external force^3,30,31^. Accordingly, the SCB thickens in response to OA-associated cartilage degeneration as a result of increased loading, which decreases the shock-absorbing ability of the bone, noted most acutely in regions peripheral to the area closest to articulation^40,41^, which may lead to an increase in microcracks and SCB lesions^38,42^. Notably, SCB thickening co-localizes with cartilage lesions, with the least thickening detected in places where the overlying cartilage is maintained^43^, which provides an indication of the regions exposed to the highest amounts of stress after injury^43,44^. Consistent with this, our results in DMM mice illustrate increased Col I expression in all regions of the superficial (Fig. 3B,D), but not the deep (Fig. 4B,D), SCB, indicating that the superficial zone experiences higher compressive stress, resulting in more drastic changes in the collagenous content. However, both the superficial and deep zones exhibit thickening of Col I fibers (Figs. 3F, 4F) in late OA, indicating overall subchondral bone stiffening.

The meniscus absorbs shock and limits force transfer to the underlying cartilage^8^, allowing for mechanical stability and supporting up to 70% of the compressive load of the knee^45^. Therefore, meniscal injuries predispose individuals to OA due to increased loading on the cartilage^46^. The menisci are wedge-shaped in cross-section, with a thicker, outer vascular region, rich in circumferentially arranged collagen I fibers, and a thin, inner avascular region, with a high abundance of collagen II fibers arranged parallel to the surface of the meniscus^9,45,47^. Thus, the outer, vascular region, which constitutes the peripheral 10–30% of the meniscus^30,48,49^, has higher capacity for healing, in comparison to the inner, avascular portion. Pathological changes in an injured meniscus include meniscal hypertrophy and mineralization, as well as neovascularization in the vascular region^48,49^. Fibrosis of the avascular portion of the menisci is seen to correlate with tissue stiffness^50,51^, which results in decreased mechanical stability and transmission of load through the joint^45,48^. Though several studies have shown increased Col I and II expression in OA menisci, architectural changes of the collagen network which occur in the meniscal extracellular matrix in response to injury are yet to be characterized^2,18,35^. Our results in DMM mice provide insight on the meniscal regions most susceptible to stress in the medial compartment. We found increased expression of Col I and Col II and increased Col I-fiber thickness in the vascular region of both the anterior (Fig. 5A–E) and posterior (Fig. 6A–E) menisci. These findings suggest increased fibrosis in the menisci of DMM mice, which occurs in response to increased load/stress on the vascular region of the meniscus that takes place in OA^46^. Moreover, the elevated expression and increased thickness of Col I fibers (Figs. 5A,C,E, 6A,C,E) contribute to meniscal mineralization and hypertrophy that typify late-stage OA^28,29,52^.

This study provides a comprehensive examination of collagenous changes in the medial compartment of the knee, which undergoes the most significant degenerative changes in OA, and highlights regional differences in each joint tissue. Future studies examining the central and lateral compartments could provide further elucidation of the changes throughout the entire knee during disease progression.

## Methods

### Study design

This study follows the ARRIVE guidelines 2.0. Sample size was determined as previously described^1^ using the G*Power 3.1 software. Based on our previous experience and studies analyzing histologic parameters affected by DMM mice in comparison to sham mice, we calculated the sample size required for each group to reach 5% significance and 0.80 power. Power analysis showed that at least 4 mice in each group are required. Thus, we increased the number of mice to 5 in the sham and DMM groups to account for any individual variances. No animals were excluded from the research group. Animals were randomized for either sham or DMM surgery. Samples collected were coded and analyzed in a blinded manner until data was obtained and quantification completed. Data were then decoded to be graphed under corresponding treatment groups, with results charted and statistical analysis performed as described. Outliers in data sets were removed using Grubb’s test for exclusion. Results were confirmed by repetition 3 times.

### Animals

Twelve-week-old C57BL/6J male mice were purchased from The Jackson Laboratories. Groups of 3–5 mice per cage were housed in standard conditions (micro-isolator cage, 12-h light/dark schedule, dry pellet food and tap water available throughout the day). All animal procedures were performed according to the National Institute of Health (NIH) Guide for the care and use of laboratory animals and approved by the Animal Care and Use Committee of Pennsylvania State University.

### Post-traumatic induction of OA through destabilization of the medial meniscus (DMM)

DMM surgeries were performed on right knees of twelve-week old C57BL/6J male mice, through transection of the meniscotibial ligament (MMTL) as previously described^53^. Briefly, mice were prepped for surgery with intraperitoneal injection of ketamine (60 mg/kg) and xylazine (4 mg/kg), and the skin of the medial knee was cleaned with betadine. A 5 mm incision was made through the skin to reveal joint structures and the patellar tendon was identified. The MMTL was transected with a #11 scalpel allowing for increased movement of the medial meniscus within the joint. To close the incision site, 4–0 silk sutures in an interrupted pattern were placed and the site cleaned with betadine. Sham surgeries were performed on the left knees, in which the same procedure was performed without disruption of the MMTL. Analgesia was provided by intraperitoneal injection of buprenorphine (0.5 mg/kg) every 12 h for 3 days. Sutures were removed after 7 days. After 12 weeks post-DMM, mice were sacrificed by whole animal perfusion (with 10% NBF). Mouse knee joints were harvested, fixed with 10% neutral buffered formalin (NBF) for one week, then decalcified in two changes of decalcification buffer (14% EDTA Tetrasodium, 1.5% glacial acetic acid in dH_2_O) over one week. Samples were then embedded in paraffin wax and serial sections of DMM and sham knee joint samples were obtained at 5 µm thickness with a rotational microtome. Sections were then used for Safranin-O/Fast Green, Picrosirius red (PSR), and immunofluorescence staining and histological analysis. Safranin-O/Fast Green images were acquired with OsteoMeasure Software (https://www.osteometrics.com/).

### Picrosirius red staining, imaging, and quantification

5 µm sections of the medial knee joint were deparaffinized and stained with picrosirius red solution for 1 h at RT. Slides were then rinsed 2 × in 0.5% acetic acid solution, then dehydrated, cleared in xylene, and mounted with resinous medium^2,3^. Slides were imaged at 10 × magnification with polarized light microscopy and were quantified using ImageJ. Images were split into red and green channels (threshold: 75–184 for green channel, 147–225 for red channel), then areas were selected and area percentage and average intensity were quantified.

### Second harmonic generation image acquisition

We have previously described the general SHG microscopy setup^13,15^. Briefly, we utilized a InSight DS + mode-locked single-box laser system, set to acousto-optic tunable filters (AOTF) to protect samples from damage. Care was taken to ensure that power setting was consistent between sample imaging. Samples were imaged with a Nikon A1 MP + Multi-Photon upright Microscope system (Nikon Instruments) with water immersion objective lens (CFI75 Apo Water 25X/1.1 LWD 2.0-mm WD) for FSHG collection, and 440/20 nm band pass filters for BSHG detection.

Safranin-O/Fast Green stained knee joint sections were used for SHG microscopy. Tibial cartilage, subchondral bone, and anterior and posterior horns of the medial meniscus from DMM and sham knee joints were imaged using SHG microscopy, in order to compare and quantify collagenous changes within specific regions of interest. Images of the tibia were acquired at anterior, middle, and posterior regions in reference to the anterior and posterior meniscal horns, for an even representation of the cartilage (radial zone of uncalcified cartilage), superficial subchondral bone (inferior to the osteochondral junction), and deep subchondral bone, immediately superior to the growth plate. Because the superficial and transitional zones in our DMM samples were too degraded to measure, we imaged the remaining uncalcified cartilage in the radial zone. The anterior and posterior horns of the medial menisci were analyzed by comparing vascular (external) and avascular (internal) regions for collagenous changes due to differences in blood supply. Three ROIs were captured from each area for a representative measure of the entire sample.

Each ROI was captured at a lower laser power to measure collagen content and at a higher laser power to measure fiber orientation and width. Using lower laser power prevented oversaturation of pixels between ROIs of any samples, while the higher laser power images visualized smaller fibers with lower expression during later analysis. Notably, care was taken to avoid oversaturation in each imaging session by utilizing the saturation map during image capture, as the width of discrete fibers are impossible to define if excess signal merges the fibers together.

### SHG image analysis

Utilizing the 3D reconstructions created from Z-stacked SHG images, analyses were performed as previously described^15^ using Volocity software (PerkinElmer, United Kingdom), to analyze collagen content, fiber width, and orientation index.

#### Collagen content

To determine the collagen content in the ROIs, volume fraction (VF) for both BSHG and FSHG signal was calculated and confirmed with immunofluorescence staining. For each ROI, volume fraction was calculated by dividing the volume in the ROI producing BSHG or FSHG signal by the total volume of the image in order to quantify the amount of signal within the ROI. Thicker fibers allow SHG signal to traverse the structure to the FSHG detector, while thinner fibers reflect SHG signals to the BSHG detector. Thus, the amount of FSHG and BSHG signal within a ROI is indicative of the amount of thick (Col I) and thin (Col II) fibers respectively. FSHG/BSHG VF ratio was then calculated as a measure of expression changes in Col I relative to Col II. Ratios from each ROI were averaged by sample, then plotted with GraphPad Prism. The FSHG/BSHG VF ratio was only calculated for cartilage because other joint tissues experienced a simultaneous increase in both Col I and II expression.

#### Fiber width

Fiber width was determined by measuring four discrete fibers per ROI by using the line tool in Volocity to draw a line perpendicular to a fiber, measuring FSHG and BSHG signal intensity against distance across the line. FSHG signal intensity v. distance was plotted using Origin software, yielding peaks of width equal to that of the fiber. For each ROI, 3–5 fibers were measured, and the resulting widths plotted in Graph Pad by region. Fiber widths were averaged by sample. Although FSHG/BSHG ratio has been commonly used as a parameter to evaluate the thickness of collagen fibers^16^, this calculation does not distinguish between collagen fibers that produce both FSHG and BSHG signal, and can only be used as a measure in locations with only one type of collagen if statements are to be made about the thickness of Col I v. Col II fibers. It therefore does not provide a concrete measurement of the fibers themselves, but rather provides a relative measure of the amount of thick and thin fibers present in a ROI.

#### Orientation index

Fiber orientation was determined using a MatLab Fast Fourier Transform (FFT) script, in order to quantify the organization of collagen fibers in the ECM. This calculation determines how aligned the fibers are within the sample, providing a numerical value for the angle at which the fibers are placed in relationship to each other (0 = perpendicular orientation, 90 = parallel orientation).

### Immunofluorescence

Immunofluorescence staining was utilized to determine localization of collagen types I and II. 5 µm sections were deparaffinized. Antigen retrieval was achieved by incubation in 0.5% Pepsin/0.5 N HCl solution at 37 °C for 30 min, followed by incubation in hyaluronidase solution (1.5 mg/1 mL 1xTBS) at 37 °C for 5 min. The slides were then permeabilized by incubation in 0.03% Triton X in 1xTBS for 30 min. Samples were blocked in 10% normal goat serum at RT for two hours, then incubated overnight at 4 °C with rabbit anti-mouse Col II antibody (ab34712, 1:100). Slides were then washed 3 times with 1X TBS, incubated with goat-anti rabbit AF568 antibody (1:100) for 1 h at RT. Then, samples were incubated in goat biotinylated Col I antibody (ab24821, 1:50) overnight at 4C, washed in 1X TBS, and incubated with Streptavidin AF647 Conjugate (S32357, 1:100) for 1 h at RT. Negative control slides for Col I and II were stained as described, but incubated O/N at 4 °C with IgGs instead of primary antibodies. All samples were mounted with DAPI Prolong Gold, cover-slipped, and imaged with a Zeiss Axio Observer Inverted Microscope at 40 × magnification. Images were acquired with Zeiss Zen Blue 2.6 edition (https://www.zeiss.com/microscopy/us/products/microscope-software/zen.html).

### Immunofluorescence staining analysis

IF stains for Col I and Col II were quantified utilizing Zen Blue imaging analysis software. In each 40 × image, 3 ROIs (235 × 235 um to replicate the magnification of the SHG images) were selected for measurement of average fluorescence intensity (pixel intensity) and area percentage (number of pixels within an image containing fluorescence) of each collagen of interest. Threshold was set specifically for each collagen in each tissue type (cartilage, SCB, and menisci fibrocartilage) to quantify fluorescence in the matrix, and was consistent throughout analysis of each sample.

### Statistical analyses

Data for each SHG and IF parameter studied was averaged per sample for n = 5 for both sham and DMM mice. Data from sham and DMM samplers were compared using unpaired Welch’s t-test. Comparison between the three regions of the cartilage (anterior, center, and posterior) was performed using one-way ANOVA. All calculations were performed using the GraphPad Prism 9.1.0 program. Graphed values are expressed as mean ± SEM.

## Supplementary Information

Supplementary Figures.

## Data Availability

The datasets used and/or analyzed during the current study available from the corresponding author on reasonable request.
