# Supplementary material for: Structural changes in the collagen network of joint tissues in late stages of murine OA
Source: Sci Rep. 2022 Jun 1;12:9159. doi: 10.1038/s41598-022-13062-y (PMC9160297; doi:10.1038/s41598-022-13062-y)
Supplement: Supplementary file 1 — Supplementary Figures. [file 41598_2022_13062_MOESM1_ESM.pptx]

## Slide 1
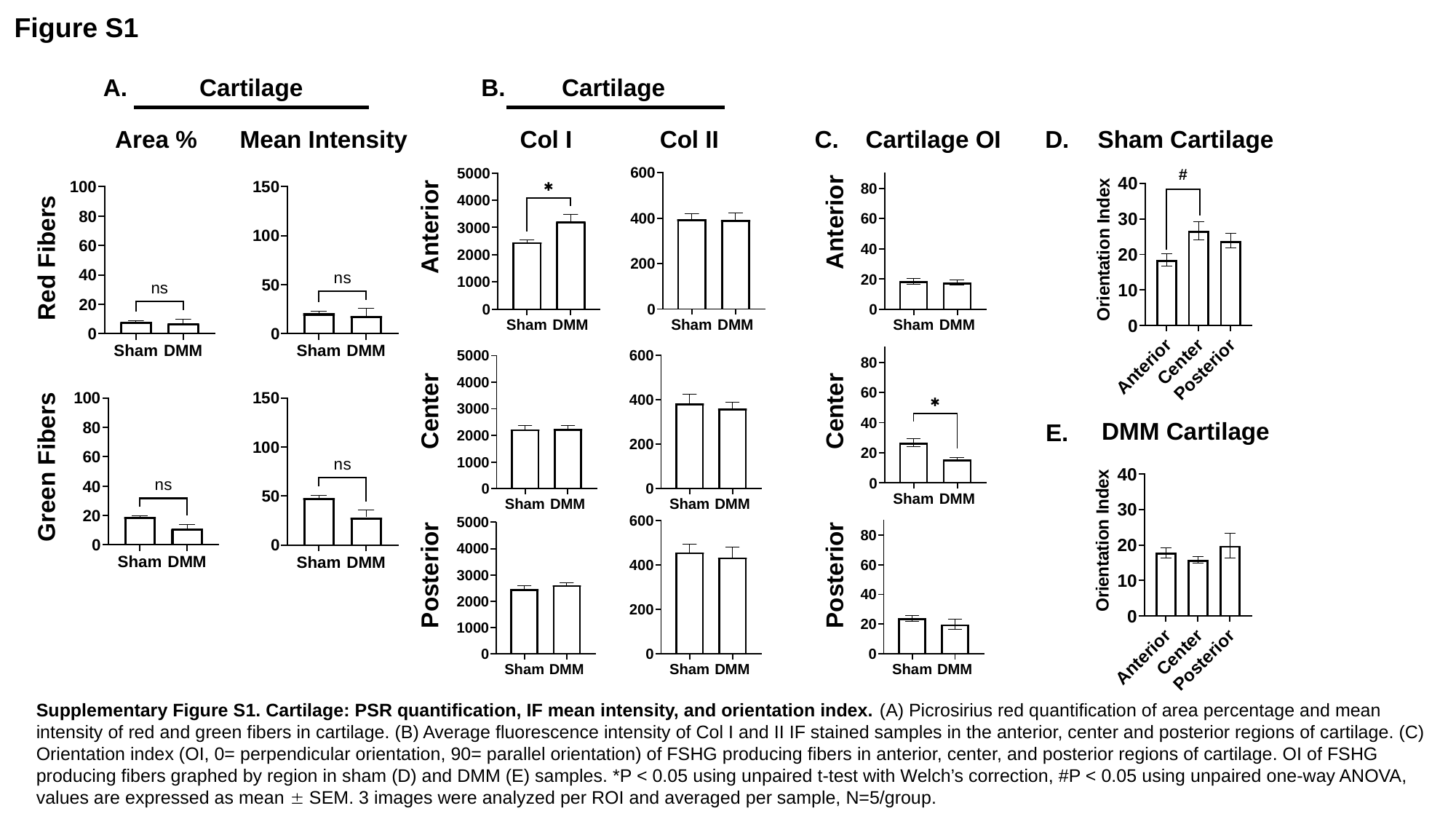

Figure S1
A.
Cartilage
B.
Cartilage
Area %
Mean Intensity
Col I
Col II
C.
Cartilage OI
D.
Sham Cartilage
Anterior
Anterior
Red Fibers
Center
Center
DMM Cartilage
E.
Green Fibers
Posterior
Posterior
Supplementary Figure S1. Cartilage: PSR quantification, IF mean intensity, and orientation index. (A) Picrosirius red quantification of area percentage and mean intensity of red and green fibers in cartilage. (B) Average fluorescence intensity of Col I and II IF stained samples in the anterior, center and posterior regions of cartilage. (C) Orientation index (OI, 0= perpendicular orientation, 90= parallel orientation) of FSHG producing fibers in anterior, center, and posterior regions of cartilage. OI of FSHG producing fibers graphed by region in sham (D) and DMM (E) samples. *P < 0.05 using unpaired t-test with Welch’s correction, #P < 0.05 using unpaired one-way ANOVA, values are expressed as mean ± SEM. 3 images were analyzed per ROI and averaged per sample, N=5/group.

## Slide 2
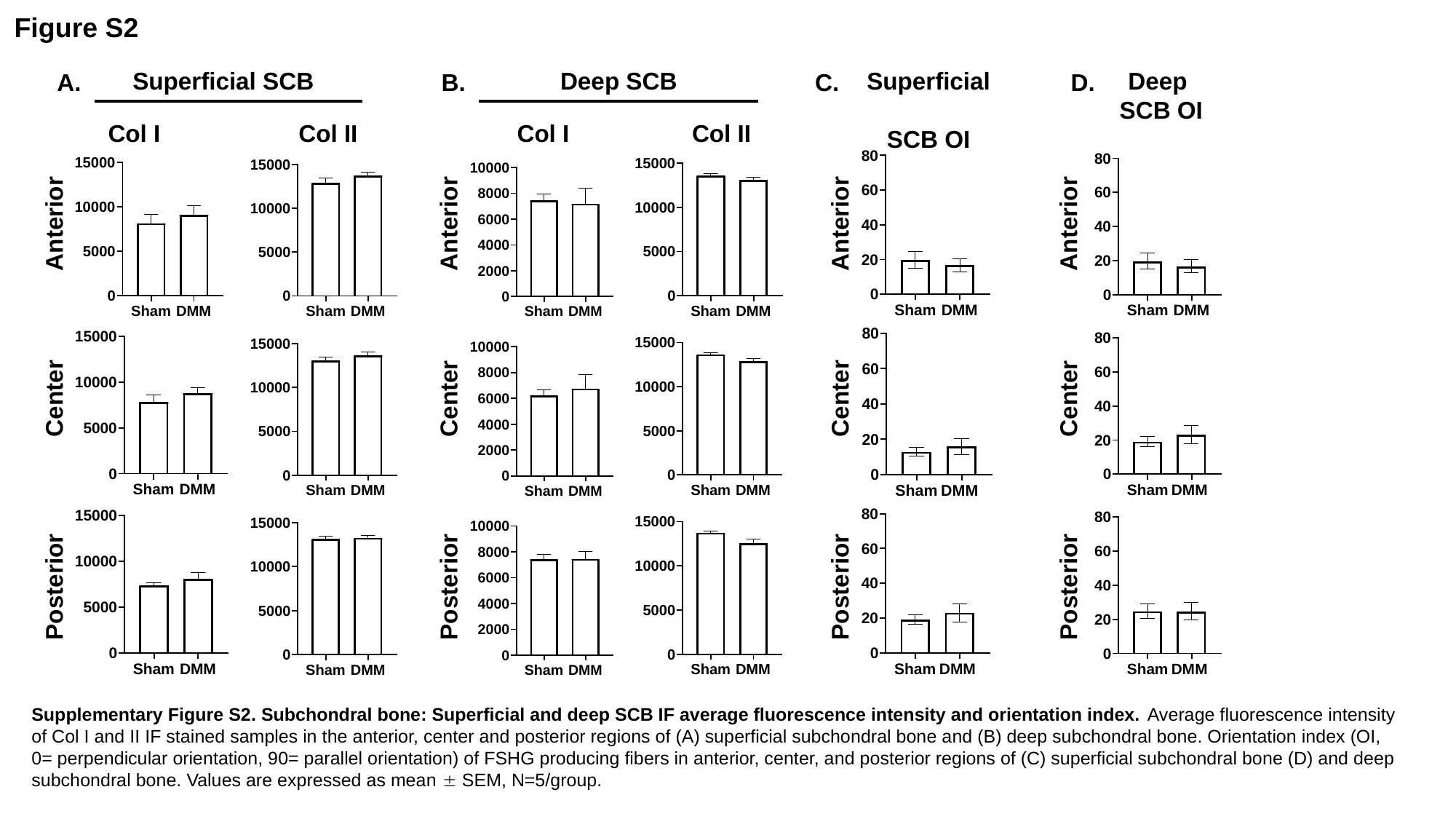

Figure S2
Superficial SCB
Deep SCB
Superficial
SCB OI
Deep
SCB OI
A.
B.
C.
D.
Col I
Col II
Col I
Col II
Anterior
Anterior
Anterior
Anterior
Center
Center
Center
Center
Posterior
Posterior
Posterior
Posterior
Supplementary Figure S2. Subchondral bone: Superficial and deep SCB IF average fluorescence intensity and orientation index. Average fluorescence intensity of Col I and II IF stained samples in the anterior, center and posterior regions of (A) superficial subchondral bone and (B) deep subchondral bone. Orientation index (OI, 0= perpendicular orientation, 90= parallel orientation) of FSHG producing fibers in anterior, center, and posterior regions of (C) superficial subchondral bone (D) and deep subchondral bone. Values are expressed as mean ± SEM, N=5/group.

## Slide 3
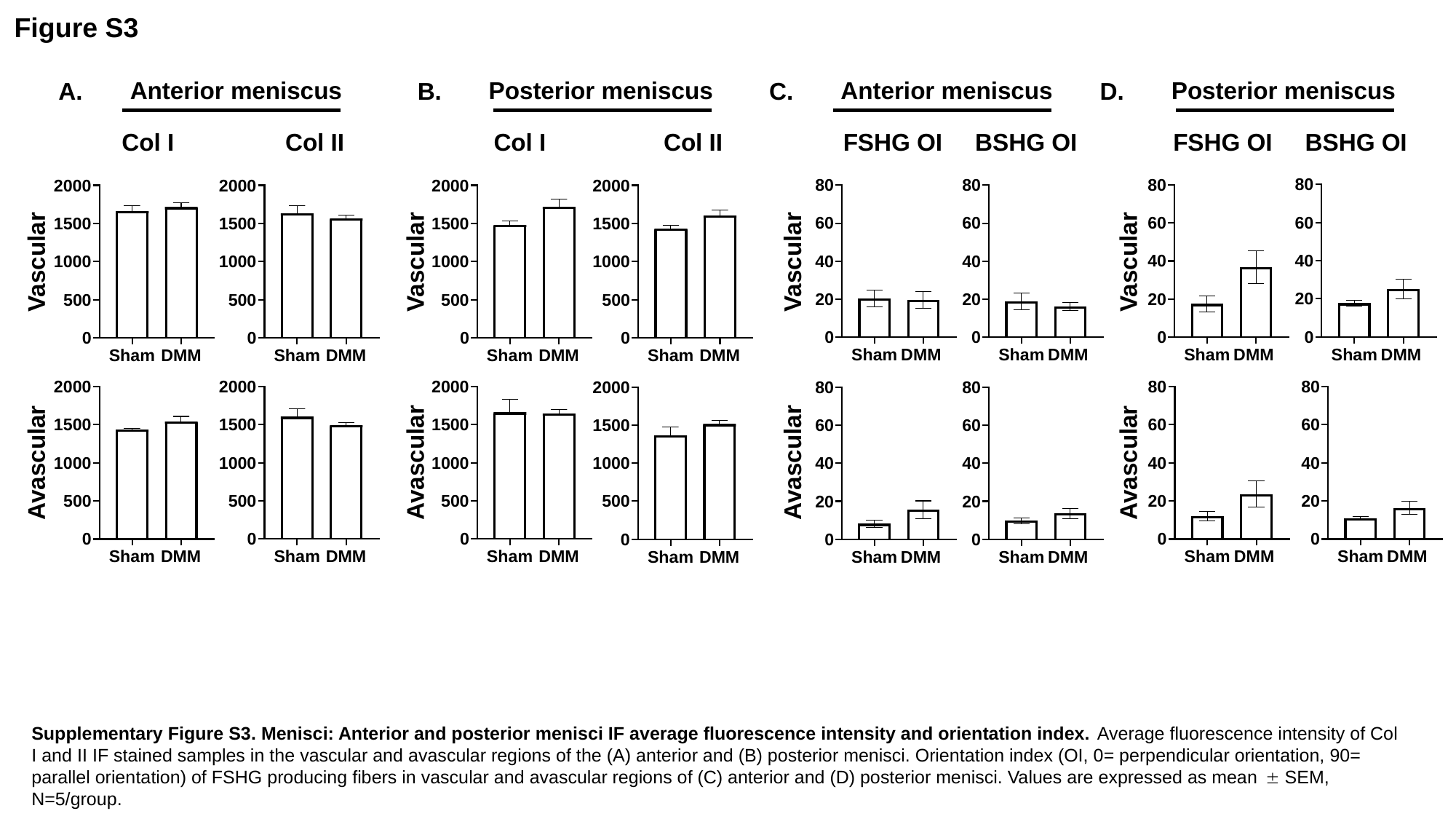

Figure S3
Anterior meniscus
Posterior meniscus
Anterior meniscus
Posterior meniscus
A.
B.
C.
D.
Col I
Col II
Col I
Col II
FSHG OI
BSHG OI
FSHG OI
BSHG OI
Vascular
Vascular
Vascular
Vascular
Avascular
Avascular
Avascular
Avascular
Supplementary Figure S3. Menisci: Anterior and posterior menisci IF average fluorescence intensity and orientation index. Average fluorescence intensity of Col I and II IF stained samples in the vascular and avascular regions of the (A) anterior and (B) posterior menisci. Orientation index (OI, 0= perpendicular orientation, 90= parallel orientation) of FSHG producing fibers in vascular and avascular regions of (C) anterior and (D) posterior menisci. Values are expressed as mean ± SEM, N=5/group.

## Slide 4
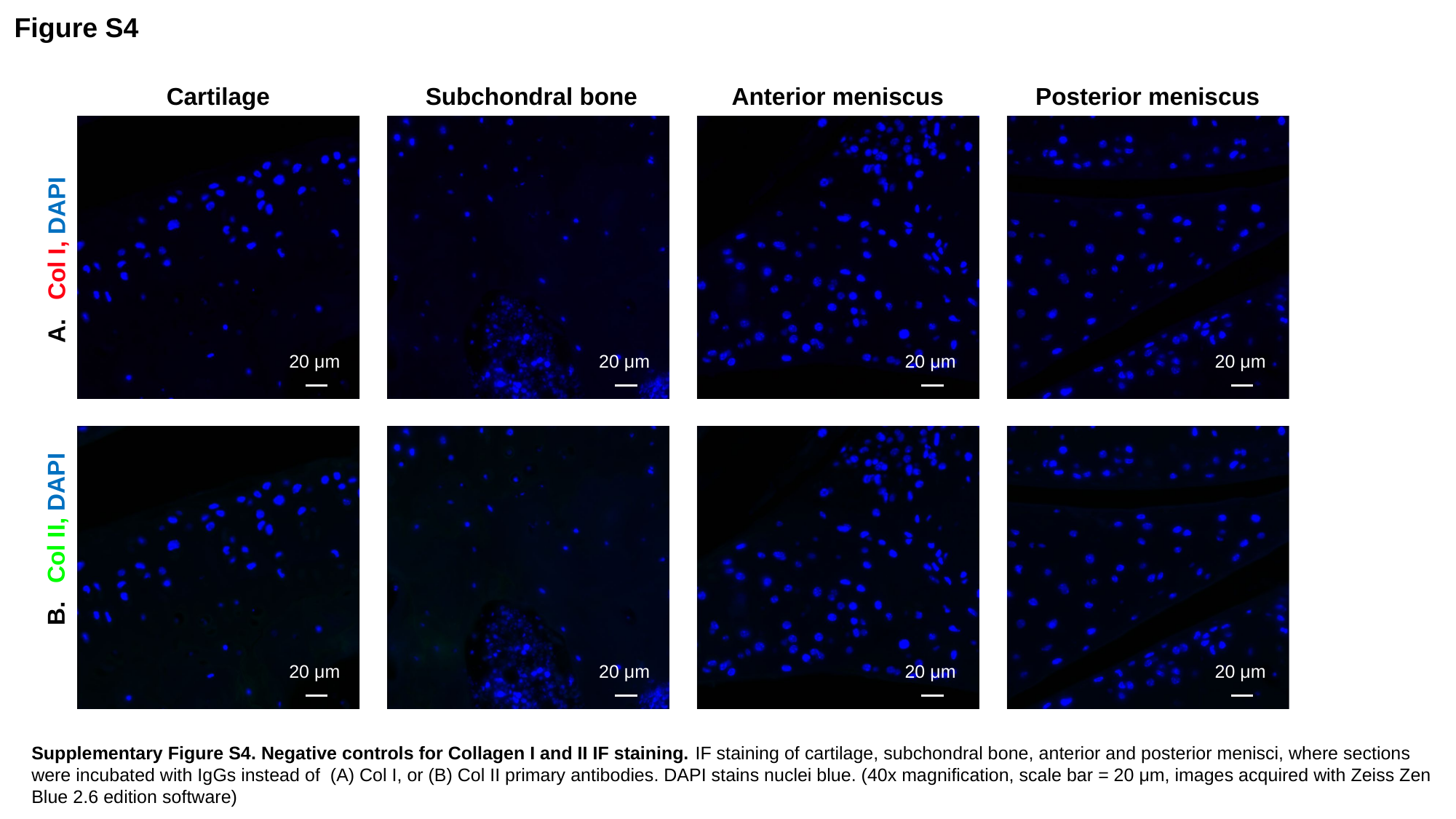

Figure S4
Cartilage
Subchondral bone
Anterior meniscus
Posterior meniscus
A.
Col I, DAPI
20 μm
20 μm
20 μm
20 μm
B.
Col II, DAPI
20 μm
20 μm
20 μm
20 μm
Supplementary Figure S4. Negative controls for Collagen I and II IF staining. IF staining of cartilage, subchondral bone, anterior and posterior menisci, where sections were incubated with IgGs instead of (A) Col I, or (B) Col II primary antibodies. DAPI stains nuclei blue. (40x magnification, scale bar = 20 μm, images acquired with Zeiss Zen Blue 2.6 edition software)
